# Method for Mid-IR Spectroscopy of Extracellular Vesicles at the Subvesicle Level

**DOI:** 10.1021/acsmeasuresciau.5c00001

**Published:** 2025-04-03

**Authors:** Nikolaus Hondl, Lena Neubauer, Victoria Ramos-Garcia, Julia Kuligowski, Marina Bishara, Eva Sevcsik, Bernhard Lendl, Georg Ramer

**Affiliations:** † Institute of Chemical Technologies and Analytics, 27259 TU Wien, 1060 Vienna, Austria; ‡ Christian Doppler Laboratory for Advanced Mid-Infrared Laser Spectroscopy in (Bio-)Process Analytics, TU Wien, 1060 Vienna, Austria; § 160631 Health Research Institute La Fe, Avenida Fernando Abril Martorell 106, 46026 Valencia, Spain; ∥ Primary Care Interventions to Prevent Maternal and Child Chronic Diseases of Perinatal and Developmental Origin Network (RICORS-SAMID) (RD21/0012/0015), Instituto de Salud Carlos III, 28029 Madrid, Spain; ⊥ Servicio de Análisis de Vesículas Extracelulares (SAVE), Health Research Institute Hospital La Fe, Avda Fernando Abril Martorell 106, 46026 Valencia, Spain; # Institute of Applied Physics, Wiedner Hauptstr. 8-10, 1040 Vienna, Austria

**Keywords:** extracellular vesicles, AFM-IR, infrared spectroscopy, nanoscale imaging, chemometrics, heterogeneity

## Abstract

Extracellular vesicles (EVs) are nanosized particles that are associated with various physiological and pathological functions. They play a key role in intercell communication and are used as transport vehicles for various cell components. In human milk, EVs are believed to be important for the development of acquired immunity. State-of-the-art analysis methods are not able to provide label-free chemical information at the single-vesicle level. We introduce a protocol to profile the structure and composition of individual EVs with the help of atomic force microscopy infrared spectroscopy (AFM-IR), a nanoscale chemical imaging technique. The protocol includes the immobilization of EVs onto a silicon surface functionalized with anti-CD9 antibodies via microcontact printing. AFM-IR measurements of immobilized EVs provide size information and mid-infrared spectra at subvesicle spatial resolution. The received spectra compare favorably to bulk reference spectra. A key part of our protocol is a technique to acquire spectral information about a large number of EVs through hyperspectral imaging combined with image processing to correct for image drift and select individual vesicles.

## Graphical abstract

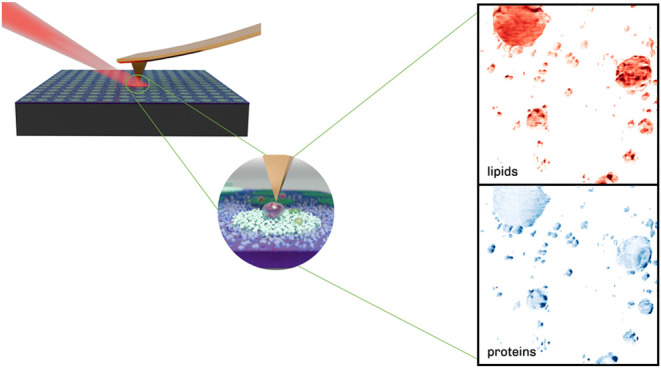

## 1 Introduction

Extracellular vesicles (EVs) are nanosized vesicles (30 nm to 10 μm)^1^ enclosed by a lipid bilayer membrane that are released by cells and are related to physiological and pathological functions.^2^ EVs are present in all biological fluids, and they contain specific cellular components, such as lipids, proteins, nucleic acids, and metabolites.^3^ They are known to release their cargo by membrane fusion to a receptor cell and are believed to be involved in intercellular communication.^4^ EVs are being explored as potential drug-delivery vectors in therapeutic applications.^5,6^ However, the physiological purpose of EVs remains largely unknown and is still being investigated.^7–9^ The analysis of EVs is challenging due to their small size and biochemical complexity.^10^ For single-vesicle characterization, techniques like nanoparticle tracking analysis (NTA), dynamic light scattering (DLS), atomic force microscopy (AFM), transmission electron microscopy (TEM), or scanning electron microscopy (SEM) have been described.^11,12^ Labeling or dyeing of the EVs can be used to get chemical information on a selected few markers and can be read out via fluorescence microscopy or flow cytometry.^11^ Label-free bulk chemical information can be recorded by using infrared (IR)-spectroscopy techniques or omics techniques.^11,13^ State-of-the-art methods provide either bulk chemical information on a large number of EVs or single-vesicle information; however, biochemical information at the single EV level is only accessible using labeling of surface markers.^1,11,12,14^

It has been previously shown that atomic force microscopy infrared (AFM-IR) is capable of acquiring mid-IR spectra of individual EVs and can thus provide label-free chemical information at the single EV level.^15^ AFM-IR is a hyphenation technique of atomic force microscopy and infrared spectroscopy, which enables the acquisition of infrared spectra at nanoscale (≈20 nm) spatial resolution, i.e., far below the diffraction limit of optical mid-IR microscopy.^16,17^ In AFM-IR, the sample is placed in a scanning probe microscope and illuminated with a pulsed, tunable infrared light. Parts of the sample that absorb the incident radiation will heat and expand. The transient thermal expansion induces oscillations in the cantilever. It has been shown that the amplitude of these oscillations is proportional to local infrared absorption.^18^ This work goes beyond the demonstration of feasibility of AFM-IR EV analysis by Kim et al.^15^ in three key steps: (1) by using tapping mode AFM-IR instead of contact mode, fragile vesicles can be measured repeatedly,^19^ (2) by performing hyperspectral imaging rather than single-point spectroscopy, a large number of vesicles can be analyzed in a time efficient manner and spectral information can be localized within vesicles, and (3) through selective immobilization of EVs instead of drop-casting from solution.

The immobilization is based on microcontact-printed anti-CD9 antibodies on a silicon substrate. These antibodies capture EVs, which are known to present CD9 antigens on their surface.^20^ This step removes the coffee ring effect that is typically seen in drop-casting deposition techniques, which leads to crowding of vesicles at the edge of the droplet and thus makes it hard to find isolated EVs in the AFM image. Furthermore, affinity capture allows us to wash away vesicles and other particles in the solution that do not express the CD9 antigen, relaxing the requirement for EV purification before deposition. A schematic representation of the microcontact-printed anti-CD9 antibody immobilization method is illustrated in Figure 1.

**Figure 1:**
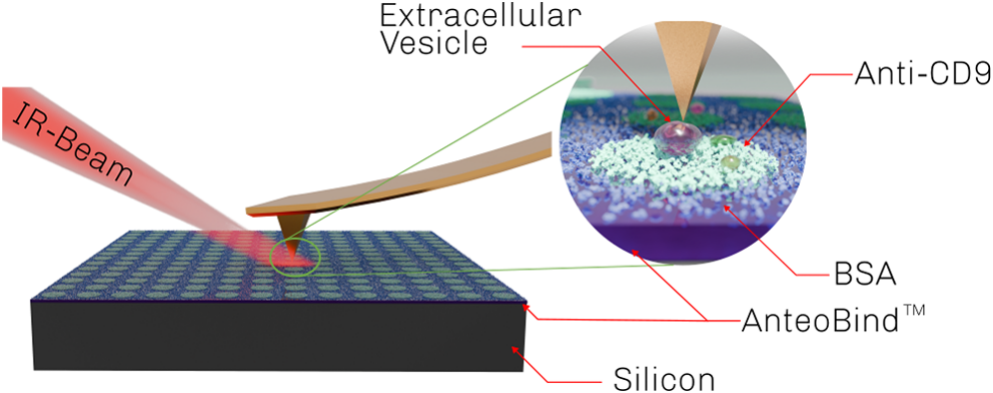
Schematic depiction of EV capturing and analysis. EVs are captured on a functionalized Si surface through antibody–antigen interaction. EVs are measured using an AFM-IR system in the top-illumination geometry.

Due to the very high spatial resolution of AFM (nanometer scale), small ambient temperature changes and piezo actuator creep, among others, introduce noticeable image translation and distortion. This thermal drift describes the unwanted movement of the AFM cantilever tip relative to the sample due to the AFM components reacting to changes of temperature.^21^ Strategies to reduce thermal drift are hardware improvements, scanning algorithms, or image reconstruction algorithms.^22^ For instance, a thermal drift-corrected cantilever,^23^ where a high-resolution silicon probe is attached to a soft silicon nitride (SiN) cantilever, is used to minimize temperature fluctuations. Alternatively, a thermostatic control system is applied to maintain a stable temperature.^24^ Additionally to these hardware solutions, techniques such as local circular scanning (LCS),^25^ where local scans are used to accurately measure the amount of drift to adjust the AFM tip, or cross-diagonal scanning combined with adaptive filtering,^22^ can be used. Another feasible strategy would be to divide the AFM image into multiple sections and perform local scans to compensate for the drift.^26^ In the scope of this article, we apply an approach based on scale-invariant feature transform (SIFT) to topography images to correct the drift in AFM-IR images. This is a key enabling step in using AFM-IR for hyperspectral imaging.

## 2 Experimental Section

### 2.1 Preparation of Extracellular Vesicles

This study was conducted in accordance with the Declaration of Helsinki, and the protocol was approved by the Ethics Committee for Biomedical Research of the Health Research Institute La Fe (Valencia, Spain; approval number 2019–289–1). A volunteer donor was enrolled at the Human Milk Bank of the University and Polytechnic Hospital La Fe (Valencia, Spain) after signing an informed consent form. HM was collected following the instructions of the hospital staff using an electric breast milk pump. A 25 mL HM aliquot from full expression was set aside and immediately centrifuged twice at 3000*g* for 10 min at room temperature for cream, cell, and platelet removal. The defatted milk was transferred to a dry tube. Tubes were placed in a freezer at −80 °C for storage. HMEVs were isolated as described elsewhere by a multistep ultracentrifugation protocol.^27^ Here, the defatted milk was again centrifuged at 3000*g* for 10 min to remove remaining milk fat and milk fat globules. The supernatant was then transferred into a 25 mL polycarbonate bottle for ultracentrifugation. After ultracentrifugation at 30,000 rpm for 2h at 4 °C, the HMEVs were retrieved as a pellet. In the final pellet, total protein (BCA-assay) and particle count (nanoparticle tracking analysis (NTA)) were determined. The protein concentration was 4.73 μg μL^–1^, and the number of particles was 6.49 × 10^8^ ± 0.376 × 10^8^ mL^–1^ with a particle size of 209.7 nm ± 7 nm. In the end, the pellet was reconstituted in 200 μL of phosphate-buffered saline (PBS) and stored at −80 °C until analysis.

### 2.2 Preparation of Substrates

The preparation of substrates followed the protocol by Lindner et al.^28^ and Schütz et al.^29^ Briefly, silicon substrates (*CrysTec*, thickness 0.525 mm, specific resistance >3000 Ωcm Orientation (100)) were plasma cleaned and then covered by 100 μL of AnteoBindBiosensor liquid for 45 min. The AnteoBind liquid was then cast off, and the substrate was rinsed with 5 mL of Milli-Q water using a pipet. AnteoBind forms a thin (a few nanometers) film on the silicon substrate which is able to immobilize proteins through coordination bonds.^30^ Next, 30 μL of a 20 μg mL^–1^ Anti-CD9 solution (Sigma-Aldrich, Anti-CD9 antibody produced in rabbit, 1 μg mL^–1^ in buffered aqueous solution) was incubated onto a PDMS-stamp. The PDMS-stamp had 3 μm circles spaced 3 μm apart in a 0.25 cm^2^ area for microcontact printing. The stamp was rinsed with 2 mL of Milli-Q water using a pipet, dried with pressurized air, and then placed onto the prepared silicon substrate for 15 min. After removal of the stamp and further rinsing with 5 mL Milli-Q water, the pattern was backfilled with 100 μL 20 μg mL^–1^ bovine serum albumin (BSA) in PBS.

### 2.3 Sample Deposition

A stick-on incubation chamber (Grace Bio-Laboratories, FlexWellIncubation Chambers, 3.5 mL × 3.5 mL Wells, 1.8 mL Depth 25 mL × 75 mL OD, Black Silicone - Adhesive One Side) was placed on the silicon substrate. 50 μL of the EV sample solutions, diluted in PBS at a ratio of 1:10, were pipetted into these chambers. The substrates, including the EV solution, were incubated for 24 h at around 7 °C. Then, the incubation chambers were removed, and the substrates were rinsed with approximately 5 mL of Milli-Q water using a pipet and air-dried before the AFM-IR measurement.

### 2.4 AFM-IR Measurements

AFM-IR measurements were performed using a commercial AFM-IR system (nano-IR 3s, Bruker) coupled to an external cavity quantum cascade laser (EC-QCL) array (MIRcat-QT, Daylight Solutions). The measured spectra covered a range from 910 to 1950 cm^–1^ at a spectral resolution of 1 cm^–1^. The cantilevers used were gold-coated and had a nominal first free resonance at 300 ± 100 kHz and a spring constant between 20 and 75 N m^–1^ (Tap300GB-G, BudgetSensors). AFM-IR measurements were carried out in tapping mode.^31^ The cantilever was driven at its second resonance frequency (*f*_2_ = 1500 kHz), and the AFM-IR signal was demodulated at the first resonance frequency (*f*_1_ ≈ 250 kHz) with a digital lock-in amplifier (MFLI, Zurich Instruments). The laser source worked on a duty cycle of up to 20% and emitted laser pulses up to 500 mW. The laser power was adjusted between 14.75 and 100% of the original power with metal mesh attenuators. The whole instrument was purged with dry air generated by an adsorbent dry air generator. Spectra were collected in triplicates using a power of up to 63.65%. AFM-IR imaging was done measuring 1000 horizontal and 512 vertical lines for an image size between 1.5 μm × 1.5 μm to 2.6 μm × 2.6 μm using a scan rate of 0.15 Hz, a laser attenuation of 63.65%, a set point of 6.3 V, and a driving strength of 1.21%.

### 2.5 Data Processing

#### 2.5.1 Image Alignment

To counteract the lateral drift and distortion between single-wavelength AFM-IR images, image alignment was employed. Since AFM-IR provides a topography image together with each infrared absorption image, we can use the sample topography for alignment: SIFT was used to detect similar features in the topography images to align the infrared absorption images accordingly in a subsequent step. SIFT recognizes features even when they are shifted, scaled, or rotated^32^ and thus allows to also correct for rotation and skew of images.

#### 2.5.2 Spectra Alignment

Recorded AFM-IR spectra were averaged by location and smoothed using an Eiler’s smoother.^33^ To correct for sample drift during acquisition, a topography image was recorded after every third spectrum. These images were then used to correct the images using the same alignment procedure as described in Section 2.5.1. This scheme works only if the thermal drift is small compared to the resolution of AFM-IR within the time it takes to record spectra and reference images (Figure S1). Within the acquisition time of 10 s for one spectrum, a drift of less than 5 nm is expected.

#### 2.5.3 Processing

A supervised classification algorithm was used to distinguish substrate (background) and sample pixels in the chemical images. Tapping mode phase information and height information were recorded with each AFM-IR image. Training sets of pixels belonging to either substrate or EVs were selected manually. These pixels were used to establish a linear discriminant analysis (LDA) classifier. This classifier was then used to identify the background and EV pixels for further analysis. Non-negative matrix factorization (NMF) was applied to EV pixels in combined absorption images to find trends in the chemical composition of EVs.

All of the data processing steps were performed using Python version 3.10.6. SIFT was used in the implementation of scikit-image version 0.22.0, and LDA and NMF were used in the implementation of scikit-learn version 1.3.1.

## 3 Results and Discussion

A common strategy in AFM-IR analysis of a new sample type is to first identify the structure and areas of interest via topography imaging and then collect a few exploratory AFM-IR point spectra at selected locations. Here, we first scanned a 5 μm × 5 μm area containing a 3 μm diameter anti-CD9 functionalized circle. A large EV (diameter ≈ 300 nm) was selected, and point spectra were collected. These spectra were found to exhibit the same absorption bands as reference spectra from bulk EVs collected by Ramos et al.^13^ (see Figure 2a). The spectra are dominated by the 1737 cm^–1^ band, which is associated with the saturated C=O stretch vibration of lipids.^13,34^ Bonds located at 1392 and 1451 cm^–1^ are associated with CH^2^ bending of lipidic acyl chains and COO- symmetric stretch, respectively.^13,34^ The 1392 cm^–1^ band maximum is slightly shifted from the 1402 cm^–1^ position described by Ramos-Garcia et al.,^13^ but this shift is negligible compared to the overall width of the band. The most important bands for the characterization of EVs are the bands associated with the amide I vibration, originating from the C=O of the protein-peptide backbone,^34^ and amide II, which is dominated by the N–H bending vibrations of the peptide groups and the C–N stretching vibrations.^34^ In this case, we can see small broad bands around 1646 cm^–1^ and around 1542 cm^–1^, which can be associated with amide I and amide II, respectively.^13,34^ The relative intensities of the C=O and amide I and II bands differ markedly from those observed in the bulk spectra. We interpret this as an effect of laser focusing and pointing present in AFM-IR. Specifically, the normalization of the AFM-IR signal is done via the total laser intensity, while the AFM-IR amplitude is proportional to the local intensity and thus depends on the size and position of the laser spot at different wavelengths. This effect can also be observed at 1520 and 1685 cm^–1^, where the baseline shows a jump because the instrument switches from one laser chip to another. It should also be pointed out that the analyzed vesicle here has a height of around 25 nm, and the lipid bilayer has a thickness of 5 to 8 nm.^35^ Thus, while the depth sensitivity of AFM-IR has recently been described,^36^ the thickness is small enough that spectra represent a full vesicle not just the outer layers.

**Figure 2:**
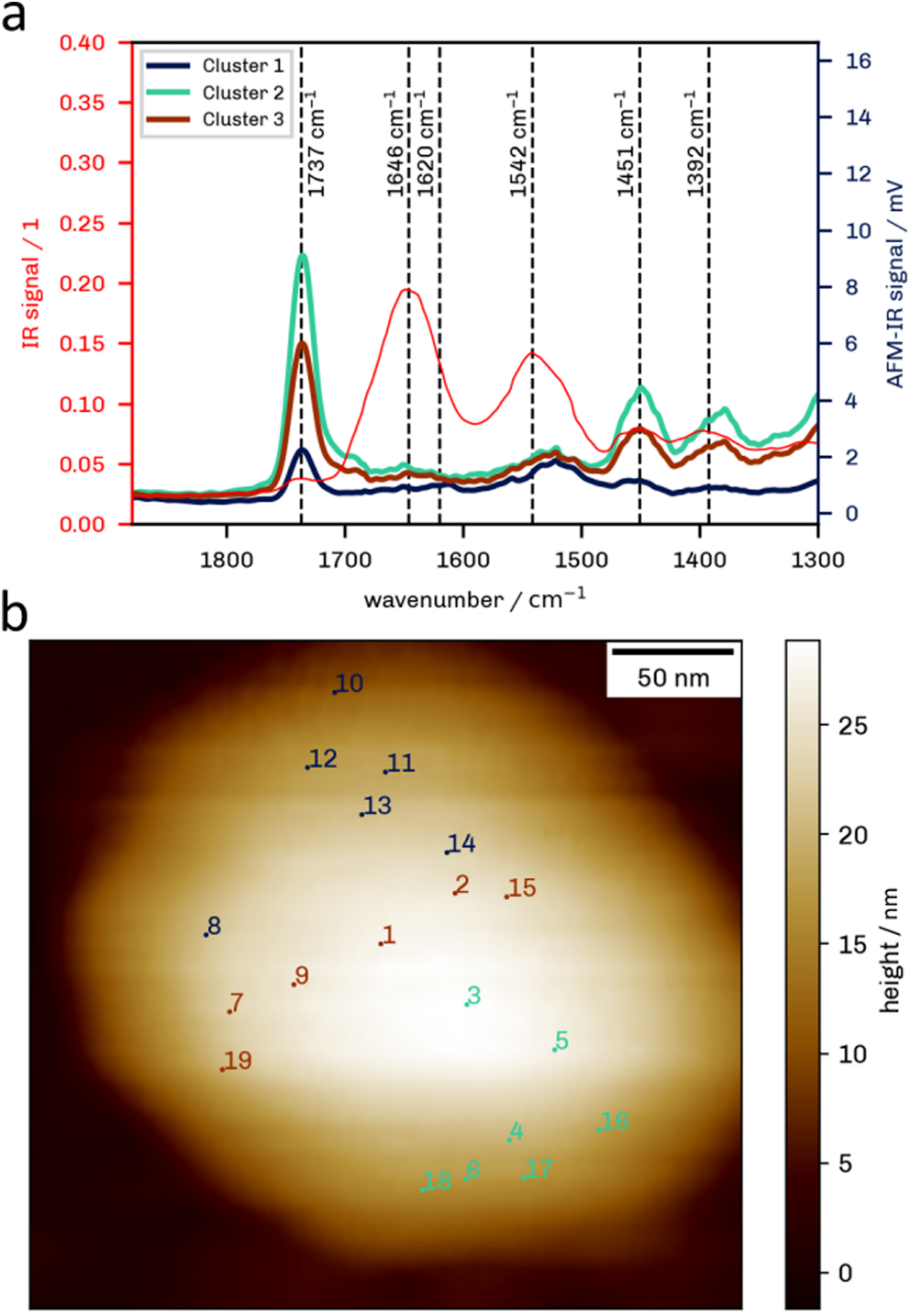
Point spectra at different locations within an EV. Mean cluster AFM-IR spectra of EVs (a) compared well to a bulk FTIR spectrum of EVs (red). AFM-IR spectra are all averages of three spectra taken at the same location and smoothed using an Eiler’s smoother (see S2). Color of spectra (see S2) corresponds to the color of the approximate sampling position markers in the AFM topography image (b).

A summary of band assignments is provided in Table 1. In general, a large difference in relative intensities was found at different locations (see Figures 2a and S2), indicating that a single vesicle shows a high compositional heterogeneity at the spatial resolution of AFM-IR. It should be noted that the substrate did not show a significant AFM-IR signal beyond a single band at 1260 cm^–1^, which can be assigned to (Si) vibrations of the substrate or cantilever (see Figure S3).^37^

**Table 1:** Identified Wavenumbers from Bulk Measurements

band position/cm^–1^	band assignment
1260	CH^3^ deformation vibration of Si-CH^3^, cantilever
1392	COO^–^ symmetric stretch
1451	CH^2^ bending of lipidic acyl chains
1542	amide II
1646	amide I
1737	saturated C=O stretch

The chemical heterogeneity within a single EV was also apparent in AFM-IR images (see Figure 3) recorded at wavenumbers corresponding to a series of absorption bands of intense spectral features in Figure 2a: 1392, 1542, 1620, 1646, and 1737 cm^–1^. Here, the intensity distributions differ depending on the absorption band. It should be noted that while large trends within the signal distributions exist, some of the strongly absorbing areas are only a few tens of nanometers in size. It should be noted that the AFM-IR signal amplitude is affected by the stiffness of the mechanical contact and other factors,^18^ such as whether the cantilever is driven at the center of the resonance or slightly off resonance.^38^ The tapping phase and tapping IR phase images corresponding to the chemical images in Figure 3 (see Figure S4) show that the phase changes across the large vesicle, indicating a change in mechanical contact stiffness and/or resonance frequency of the cantilever. Thus, even though the signal at the upper side of the vesicle is lower than at the lower side, this does not indicate a change in absorption. A similar ”half-moon effect” has also been observed in spherical structures by other researchers.^39^

**Figure 3:**
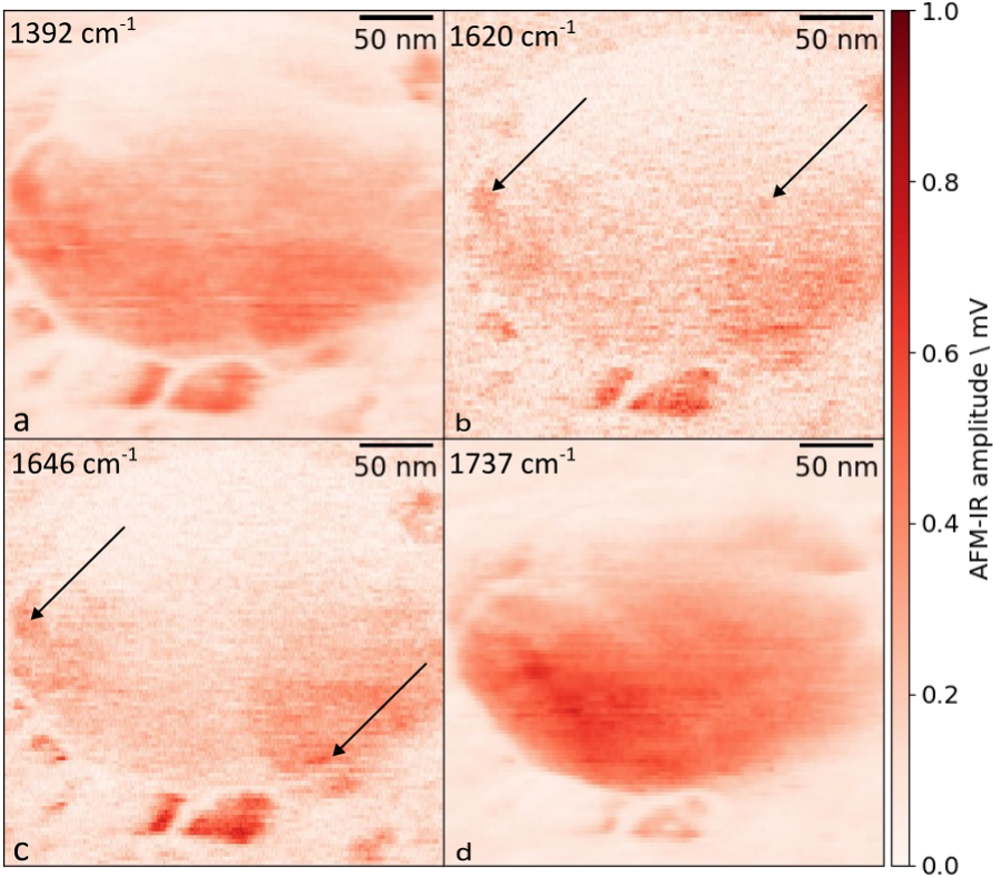
Raw AFM-IR maps of EVs taken at (a) 1392 cm^–1^, (b) 1620 cm^–1^, (c) 1646 cm^–1^, and (d) 1737 cm^–1^. The images show high location dependence of the AFM-IR signal within the EV, marked by the arrows, and large differences in distribution at different wavenumbers. The corresponding topography image is found in Figure 2b.

This chemical heterogeneity poses a problem when using single-point spectra for the characterization of chemical differences between different EVs: in order to accurately capture the composition of a single EV, a spectral grid at the spatial resolution of AFM-IR, i.e., 10 to 20 nm, would be required. This not only would make the analysis of a single EV forbiddingly cumbersome but would also cause issues of positioning errors: as AFM exhibits a slow thermal drift in position (typically in the range of nanometers per minute), taking a larger number of single spectra leads to increasing uncertainty of the actual position. This can be partially overcome by taking a topography image after every few spectra to correct for this drift as described above at the cost of increasing acquisition time.

Therefore, in this work, we collected high-pixel resolution AFM-IR images at a series of selected wavelengths (or ”marker bands”). For brevity’s sake, we refer to the fast scanning direction as ”*x*” and the slow scanning direction as ”*y*”. Furthermore, *y* was along the direction of the cantilever, and *x* was orthogonal to it. The topography channel of each image is then used to determine the drift between images and correct for it using SIFT. Results of this process are shown in Figure 4, and the full results are shown in S5.

**Figure 4:**
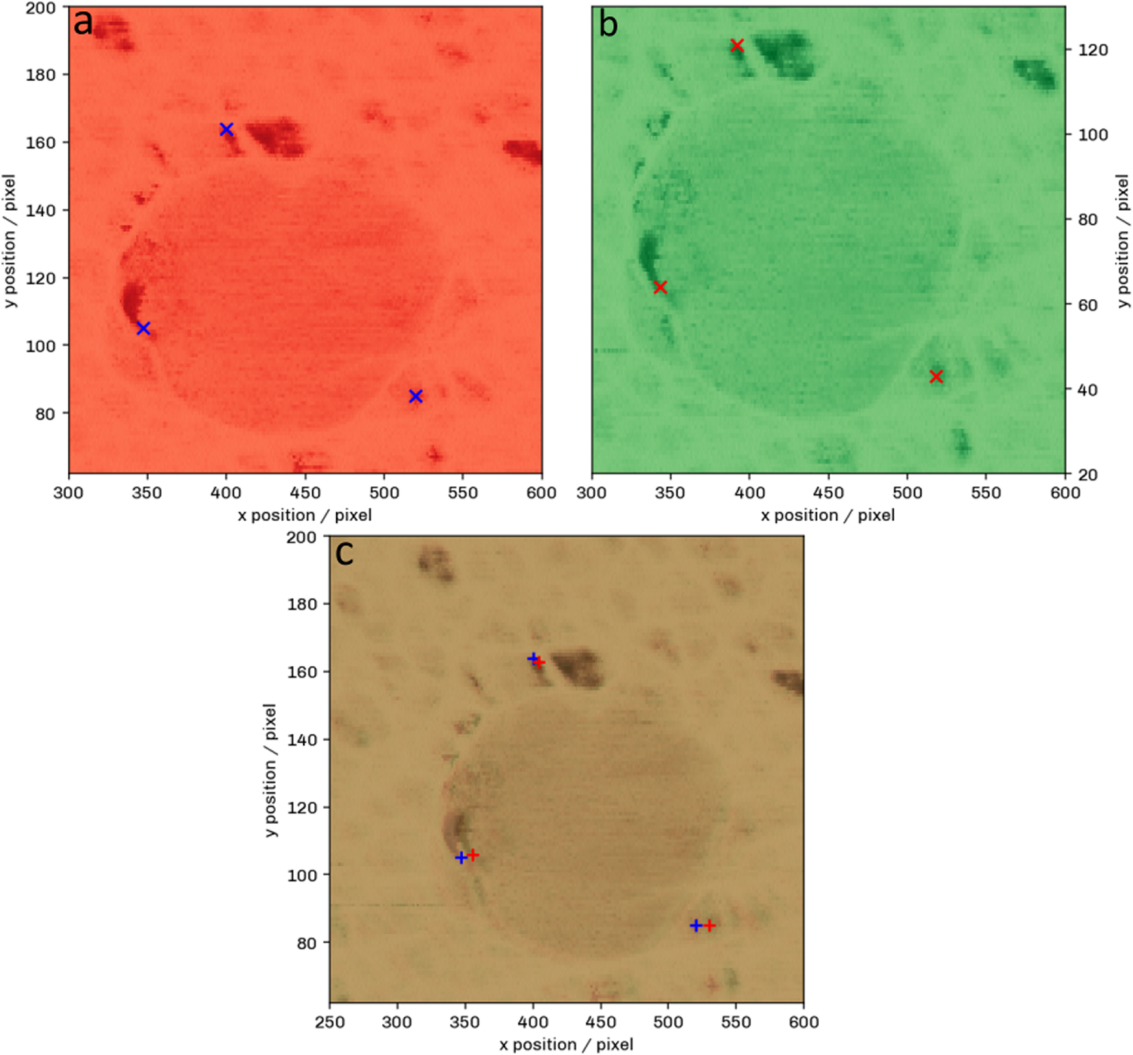
Three markers chosen on the topography reference image (a) and the compared topography image (b). The SIFT algorithm was used to determine the drift between the images, and the images were overlaid in part (c). All of the markers from parts (a, b) were projected on the overlaid image (c), and the difference can be observed. We found a maximum difference of ± 12.9 nm in *x* and ± 9.3 nm in *y*.

The performance of SIFT alignment of AFM-IR images was evaluated by manually determining the pixel coordinates of distinctive features on images and their offsets between images before and after correction. The SIFT algorithm was able to correct the drift with a maximum error of ± 12.9 nm in the *x* direction and ± 9.3 nm in the *y* direction. Table 2 shows the difference in the positions for points before and after the SIFT correction.

**Table 2:** Root Mean Square Error of Each Image in *x* and *y* Directions before and after the SIFT Correction

	RMSE *x*/nm		RMSE *y*/nm	
image	before	after	before	after
1	7.9	11.6	123.1	1.7
2	356.1	8.4	157.6	7.2
3	303.9	1.1	602.1	9.3
4	805.5	12.9	494.2	1.7
5	715.0	6.7	377.9	6.3
6	671.5	5.0	309.6	4.8

Once the full set of images has been recorded and drift-corrected, the AFM-IR images are stacked to generate a single hypermap data set, which can be evaluated using a range of chemometric techniques. In this work, the image size was chosen such that a larger number (here ≈30) of EVs was covered within a single image, parallelizing the analysis (see Figure 6). By setting up the AFM-IR instrument to automatically collect a series of images (i.e., one per marker band), this process can be carried out without user interaction after the initial setup. Figure 5 shows a topographic overview of the scanned area.

**Figure 5:**
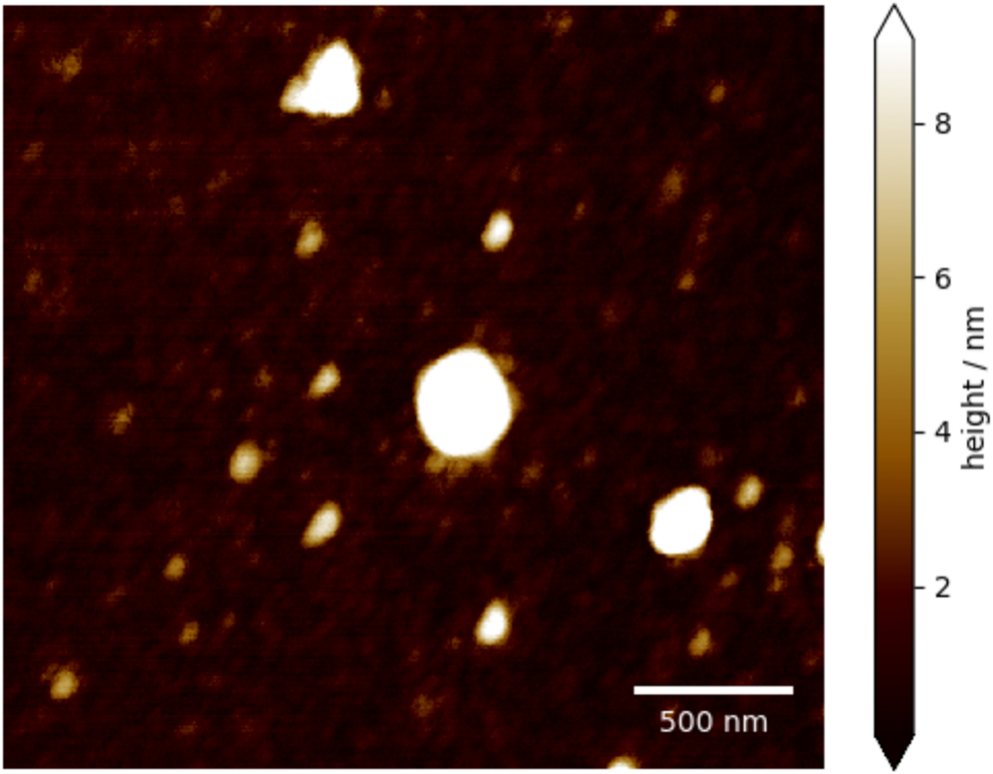
Topography image of the area, where the AFM-IR images were taken from.

NMF is used in exploratory data analysis to identify patterns by dimensionality reduction. In the case of spectroscopy, NMF can be thought of as decomposing the matrix of all sample spectra **V** (with one spectrum per row) into a product of two matrices **V** = **W H**, whereby **H** contains row-wise spectra of ”pure” components and **W** contains row-wise contributions of said components to each experimental spectrum. The number of components for NMF was selected by using principal component analysis (PCA). The first four principal components (PCs) explained 99% of the total variance (see Figure S6); hence, four PCs were used for NMF (see Figure 6). When mapping the contributions of the components onto the pixels, they largely showed smooth changes, which is a good indication that they correspond to the changes in the AFM-IR signal rather than noise. It should also be noted that the data is normalized to the band of 1260 cm^–1^ before performing NMF; thus, the before-mentioned effect on the IR images should not have a big effect on the analysis.

**Figure 6:**
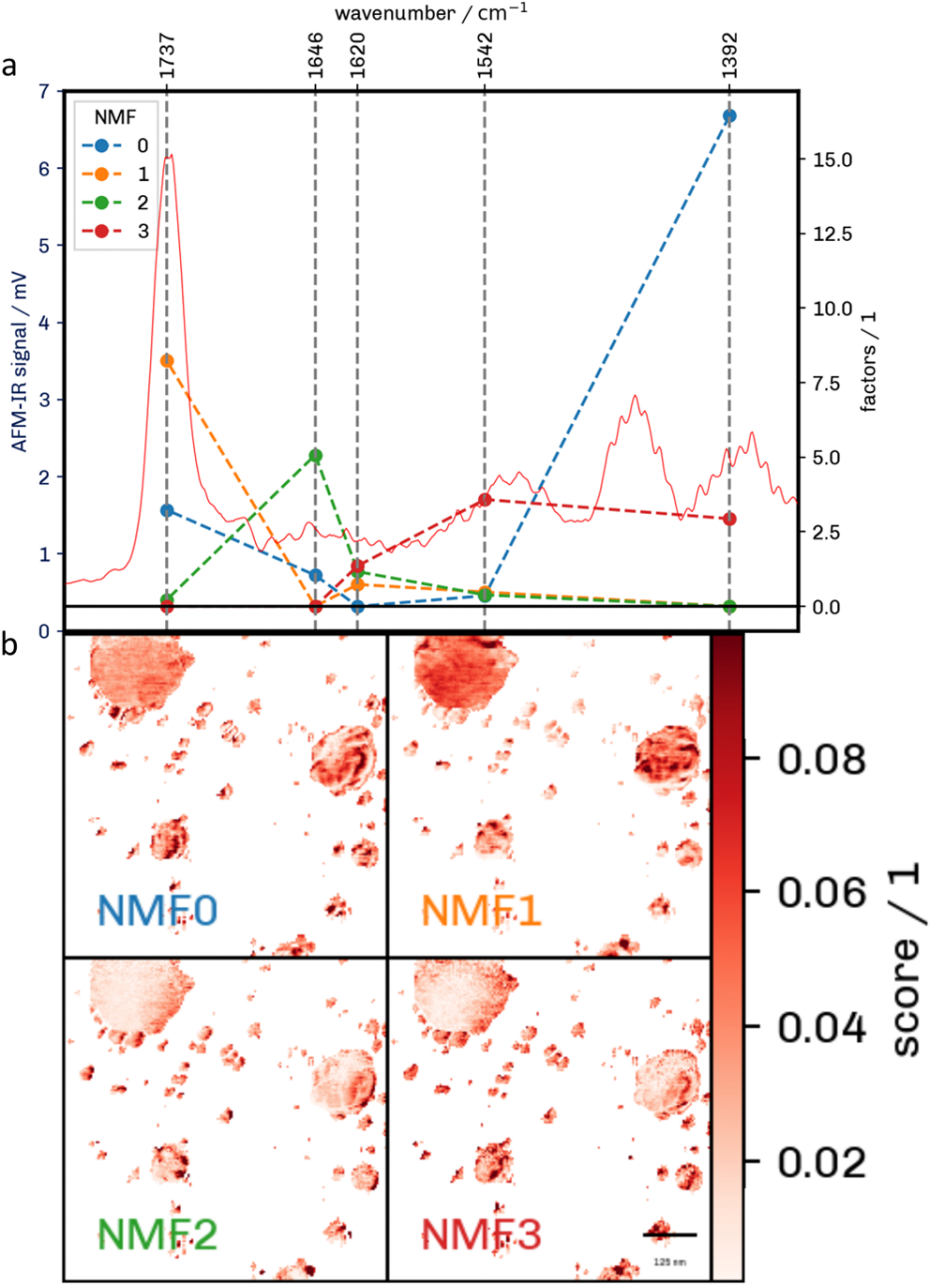
(a) Loading plot of the NMF analysis and the corresponding mean AFM-IR spectrum. (b) Score plot of the identified pixels.

The loading values (Figure 6) describe the relationship between the components and the AFM-IR signals. A higher value indicates that this wavenumber contributes more to the score of the corresponding NMF component. In the zeroth component (blue), high values can be seen at 1392 and 1737 cm^–1^. This component likely corresponds to the EV membrane, as it is distributed evenly across the EV surface area. Additionally, it is known that the membrane consists of a lipid bilayer. This is further supported, as the value at 1737 cm^–1^, which is assigned to the saturated C=O stretch, is relatively high. The highest value at 1392 cm^–1^ is likely showing contributions from lipids and proteins, strengthening the idea that this component describes the lipid bilayer. The first component (orange) shows a high value of 1737 cm^–1^. This component likely corresponds to the membrane, as it is evenly distributed across EVs with a few hotspots. Additionally, the small contribution from 1620 cm^–1^ also shows some protein in these areas. The second component (green) shows high values at the amide I band and a small value at the 1737 cm^–1^ band. This component can be used to identify the location of proteins within individual EVs. Again, some areas in the score plot have a higher value, so it is possible to identify the ”hotspots” of content in the vesicles. Additionally, these hotspots point toward α-helix or disordered protein structure. The third component (red) shows high values at 1392, 1542, and 1620 cm^–1^, whereas the signal at 1646 and 1737 cm^–1^ cannot be observed. The component has a small contribution to almost all pixels inside of EVs and some small areas where a much higher value is observed. These could point toward protein cargo that has aggregated due to the desiccation of the EVs on the substrate. It should also be noted that the value at 1620 cm^–1^ points toward the β-sheet protein structure.^40,41^ To view if a trend can be observed in the composition of vesicles, we studied the relationship between vesicle composition and mean NMF contribution per vesicle (see Figure S7a). There is no apparent trend or relationship for size and NMFs for vesicles <60 nm diameter/radius. For larger vesicles, a relationship between size and NMF may exist, but for practical reasons, only a few such large vesicles appear in the data set. The NMFs appear to follow a unimodal distribution with few outliers (see Figure S7b).

## 4 Conclusions

In this work, we demonstrated chemical analysis of individual EVs using AFM-IR in a way that allows chemical characterization at the nanoscale. We implemented a new protocol to obtain label-free chemical information from single EVs. We were able to immobilize EVs on an AFM-IR compatible substrate via anti-CD9 antibodies and were able to analyze them via the implementation of machine learning algorithms. Single EVs could be described separately, and the high heterogeneity within a single vesicle and within a whole sample set was determined. In comparison to other works, this protocol only needs very few wavenumbers to work but is still able to provide a comprehensive picture of the sample without requiring user input to select measurement spots for spectra. This enables a faster analysis of the EV samples without cherry-picking. This work shows that for heterogeneous nanoscale samples, only through hyperspectral imaging and image registration can a full picture of the distribution of components be acquired by AFM-IR.

## Supplementary Material



## Data Availability

The data underlying this study are openly available in Zenodo at DOI: 10.5281/zenodo.14514540.

## Notes

Shift along the *x*- and *y*-axes after three single-point spectra; tap IR phase showing the ”half-moon” shape; alignment of the taken images using the SIFT algorithm; scores plotted against the size of the vesicle (PDF)
