# Supplementary material for: Method for Mid-IR Spectroscopy of Extracellular Vesicles at the Subvesicle Level
Source: ACS Meas Sci Au. 2025 Apr 3;5(4):469–76. doi: 10.1021/acsmeasuresciau.5c00001 (PMC12371597; doi:10.1021/acsmeasuresciau.5c00001)
Supplement: Supplementary file 1 [file tg5c00001_si_001.pdf]

Supporting Information: A method for mid-IR spectroscopy of extracellular vesicles at the sub-vesicle level

Nikolaus Hondl,[†] Lena Neubauer,[†,‡] Victoria Ramos-Garcia,[¶] Julia Kuligowski,[¶,§,∥] Marina Bishara,[⊥] Eva Sevcsik,[⊥] Bernhard Lendl,[†] and Georg Ramer[*,†,‡]

†*Institute of Chemical Technologies and Analytics, TU Wien, 1060, Vienna, Austria*

‡*Christian Doppler Laboratory for Advanced Mid-Infrared Laser Spectroscopy in (Bio-)process Analytics, TU Wien, 1060, Vienna, Austria*

¶*Health Research Institute La Fe, Avenida Fernando Abril Martorell 106, 46026 Valencia, Spain*

§*Primary Care Interventions to Prevent Maternal and Child Chronic Diseases of Perinatal and Developmental Origin Network (RICORS-SAMID) (RD21/0012/0015), Instituto de Salud Carlos III, Madrid, Spain*

∥*Servicio de Análisis de Vesículas Extracelulares (SAVE), Health Research Institute Hospital La Fe, Avda Fernando Abril Martorell 106, 46026 Valencia, Spain*

⊥*Institute of Applied Physics, Wiedner Hauptstr. 8-10, 1040 Vienna*

E-mail: georg.ramer@tuwien.ac.at

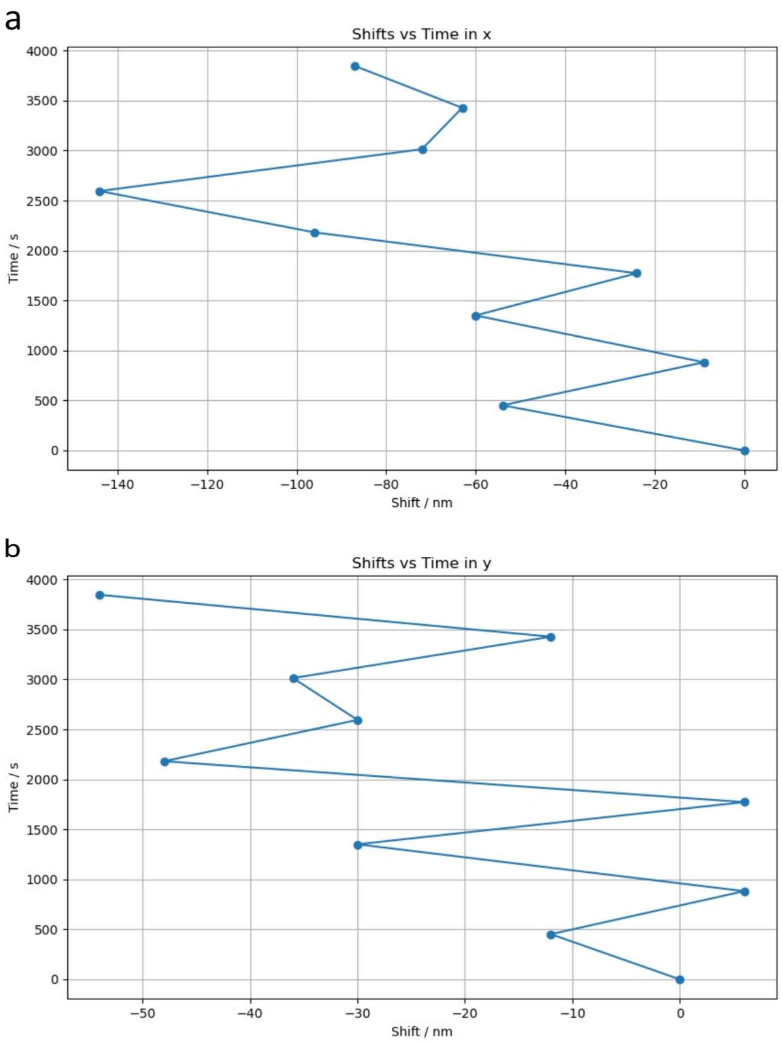

Figure S1: **Shift along the x and y axis after three single point spectra** a and b show the shift along the x and y axis after three single point spectra and a new topography image were taken

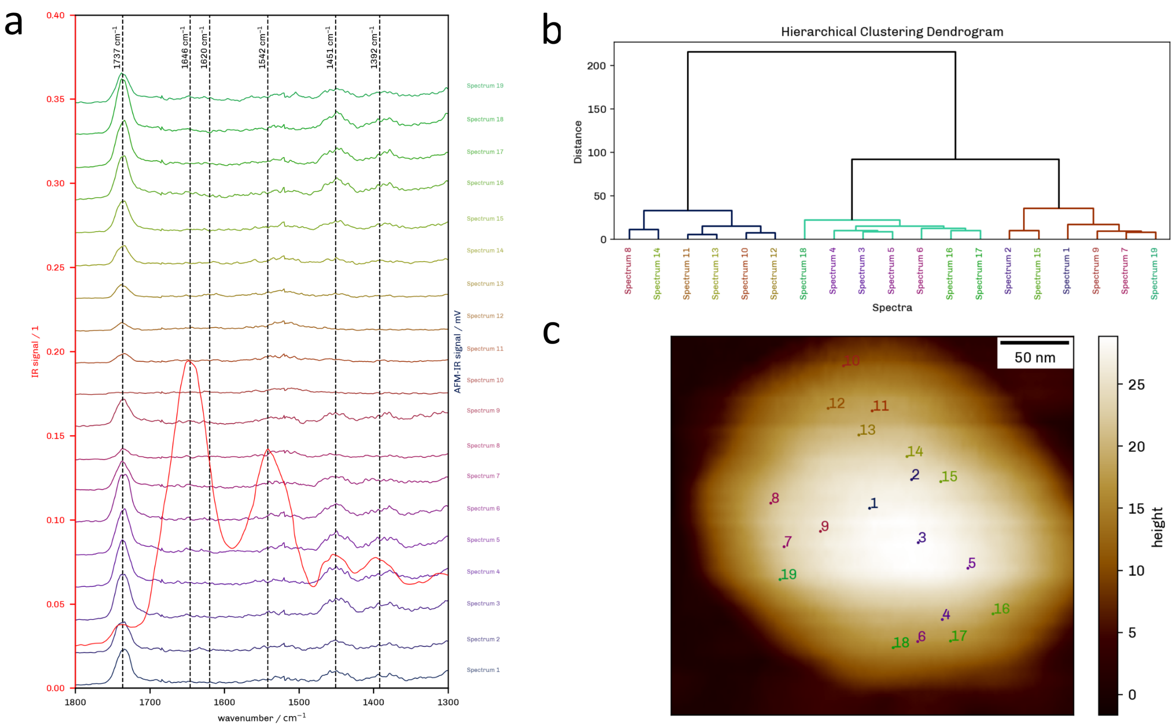

Figure S2: **(a) Stacked spectra (b) HCA of the spectra and (c) Heightmap with the position of each spectrum**

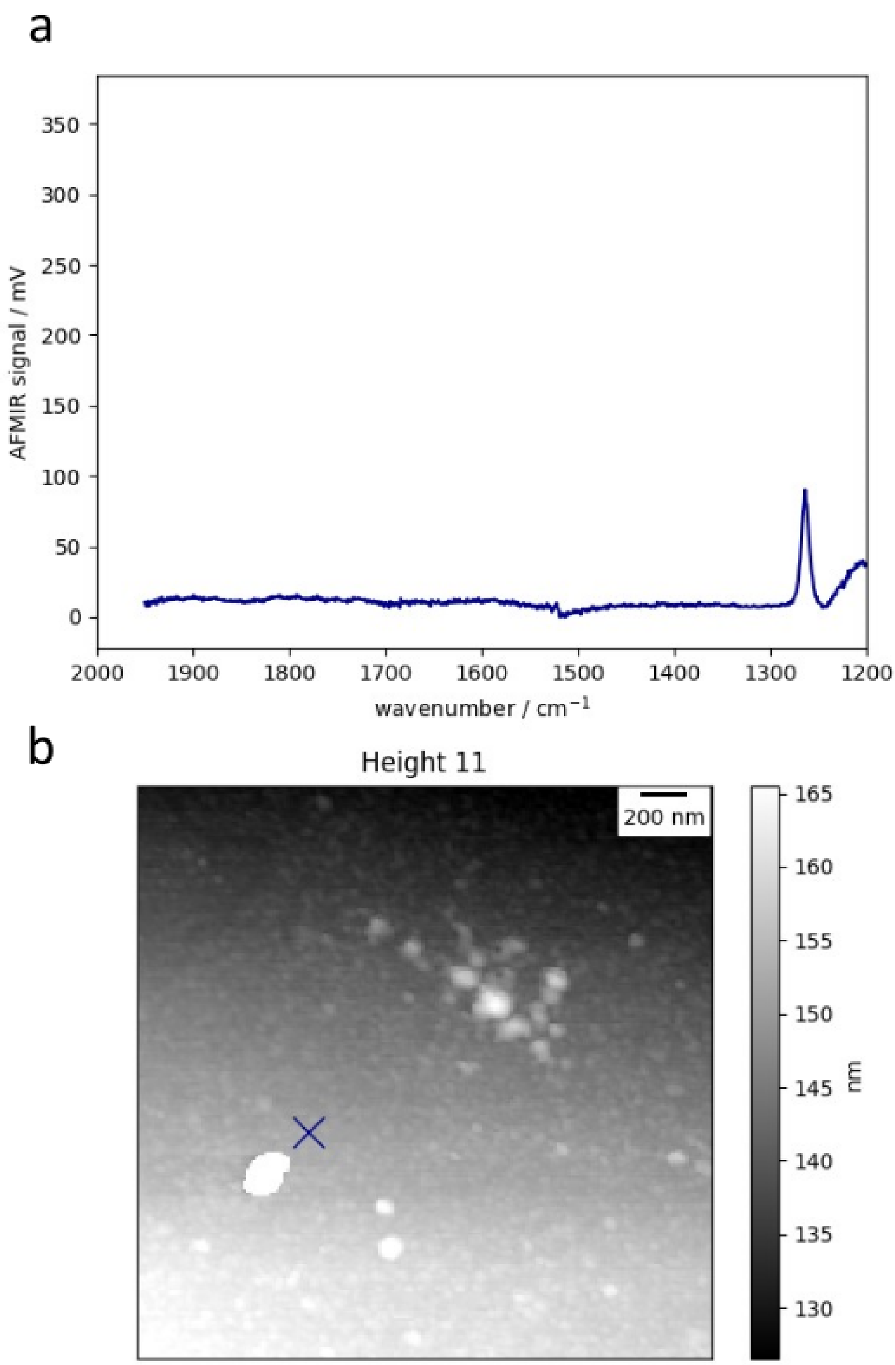

Figure S3: **Point spectrum of the substrate with AnteoBind™Biosensor** AFM-IR spectra of AnteoBind™Biosensor (a, blue) smoothed using a Savitzky-Golay filter (20 points, second order). (b) sampling position marker in the AFM topography image

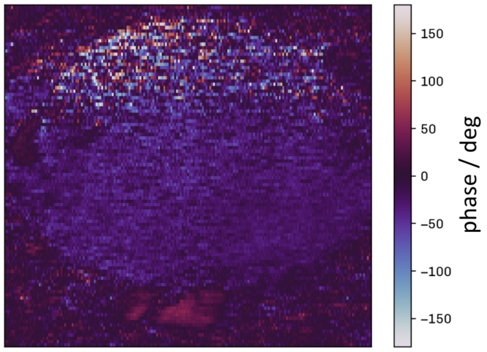

Figure S4: **Tap IR phase showing the "half-moon" shape.**

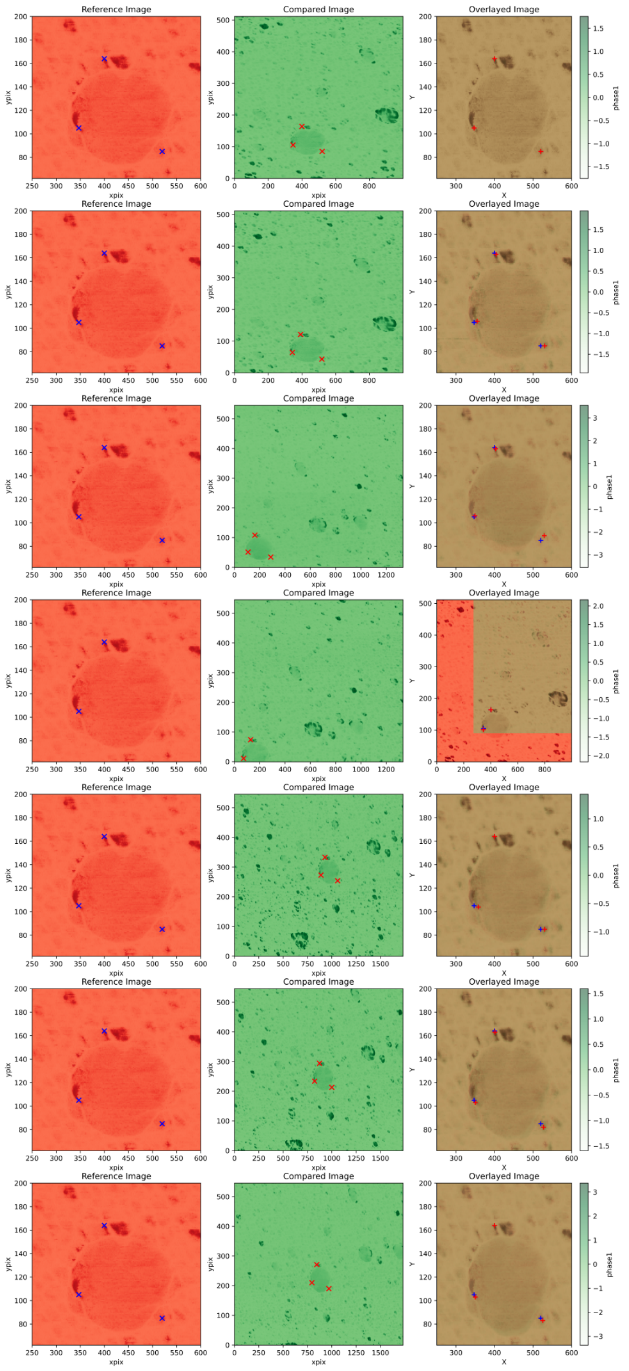

Figure S5: **Alignment of the taken images using the SIFT algorithm** Using the first image as reference image, all the other images have been compared to it. The overlayed image shows, how well the SIFT algorithm worked.

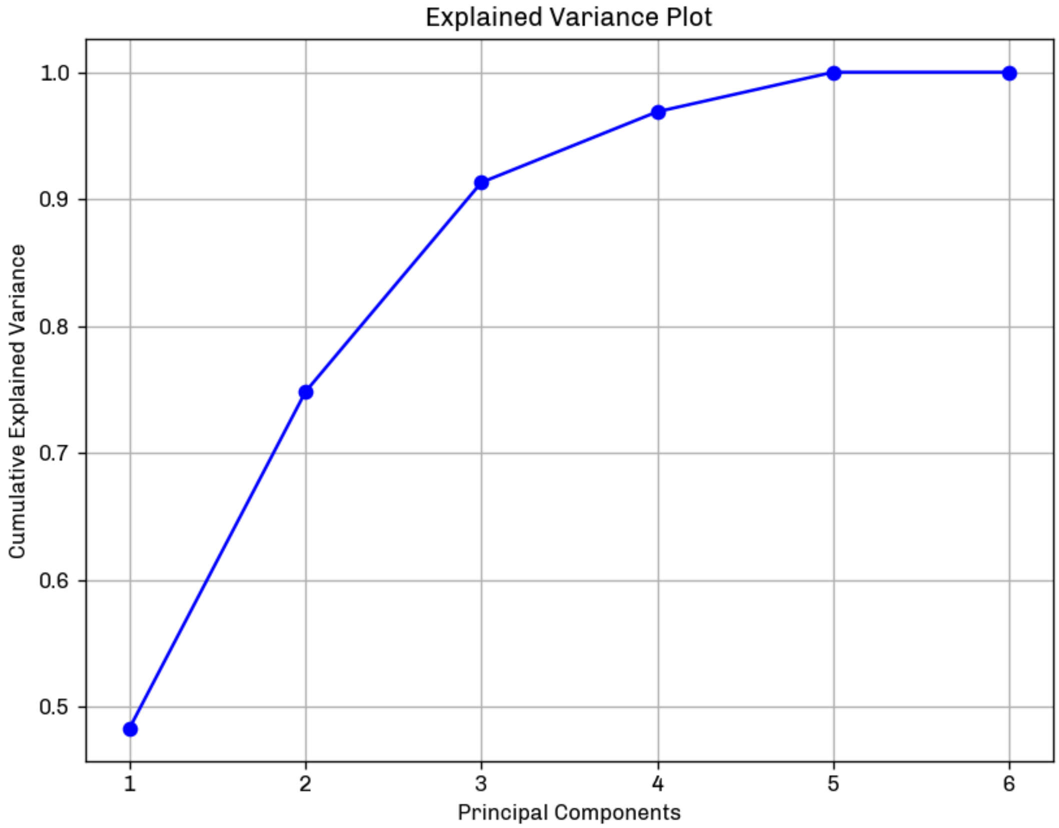

Figure S6: **Explained Variance of the PCA** Only the first four principal components were considered, as they contained the most meaningful information.

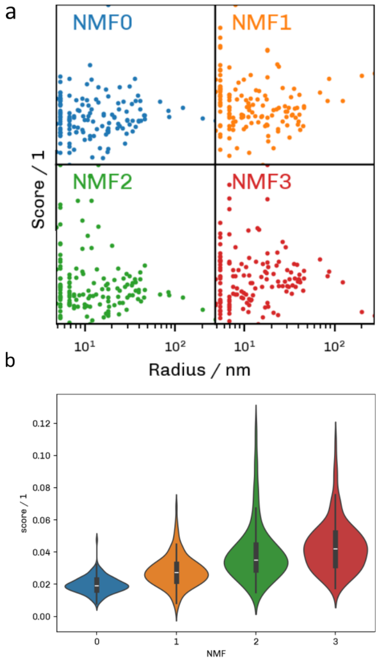

Figure S7: **(a) Scores plotted against the size of the vesicle. While the small vesicles appear not to show any trend with increasing size, but there seems to be a trend for larger (radius > 60 nm) vesicles, where NMF0, NMF2 and NMF3 tend to go down and NMF1 tends to go up. (b) Distribution of NMFs shown as violin plots. All distributions appear unimodal with few outliers.**
